# Mental Health in Individuals with a History of Mental Disorder during COVID-19-Pandemic - Preliminary Results of the National Cohort Study in Germany

**DOI:** 10.1192/j.eurpsy.2022.955

**Published:** 2022-09-01

**Authors:** S.G. Riedel-Heller, A. Pabst, J. Stein, H. Grabe, M. Rietschel, K. Berger

**Affiliations:** 1 University of Leipzig, Faculty of Medicine, Institute Of Social Medicine, Occupational Health And Public Health (isap), Leipzig, Germany; 2 University Medicine Greifswald, Department Of Psychiatry And Psychotherapy,, Greifswald, Germany; 3 Medical Faculty Mannheim, Central Institute Of Mental Health, Department Of Genetic Epidemiology In Psychiatry, Mannheim, Germany; 4 University of Münster, Institute Of Epidemiology And Social Medicine, Münster, Germany

**Keywords:** Vulnerable Groups, COVID19, Longitudinal study, Depression

## Abstract

**Introduction:**

Research of COVID-19-Pandemic mental health impact focus on three groups: the general population, (2) so called vulnerable groups (e.g. individuals with mental disorders) and (3) individuals suffering COVID-19 including Long-COVID syndromes.

**Objectives:**

We investigate whether individuals with a history of depression in the past, react to the COVID-19 pandemic with increased depressive symptoms.

**Methods:**

Longitudinal Data stem from the NAKO-Baseline-Assessment (2014-2019, 18 study centers in Germany, representative sampled individuals from 20 to 74 years) and the subsequent NAKO-COVID-Assessment (5-11/2020). The sample for analysis comprises 115.519 individuals. History of psychiatric disorder was operationalized as lifetime self-report for physician-diagnosed depression. Depressive symptoms were measured with the PHQ 9.

**Results:**

Mean age of the sample at baseline was 49.95 (SD 12.53). It comprised 51.70 women; 14 % of the individuals had a history of physician-diagnosed depression. Considering a PHQ-Score with cut-off 10 as a clinical relevant depression, 3.65 % of the individuals without history of depression and 24.19 % of those with a history of depression were depressed at baseline. The NAKO-COVID-Assessment revealed 6.53 % depressed individuals without any history of depression and a similar rate of 23.29 % in those with history of depression.

**Conclusions:**

In contrast to that what we expected, individuals with a history of a physician-diagnosed depression, did not react with increasing depressiveness during the first phase of the pandemic in Germany. Several reasons could be discussed. Whether there medium and long-term impact remains open.

**Disclosure:**

No significant relationships.

